# Composition consistency as a critical quality attribute for material extrusion additive manufacturing

**DOI:** 10.1557/s43579-026-00936-9

**Published:** 2026-02-17

**Authors:** Laurel Hilger, Alexandra Marnot, Blair Brettmann

**Affiliations:** 1 School of Materials Science and Engineering, Georgia Institute of Technology, Atlanta, GA 30332 USA; 2 School of Chemical and Biomolecular Engineering, Georgia Institute of Technology, Atlanta, GA 30332 USA

**Keywords:** Additive manufacturing, 3D printing, Extrusion, Polymer

## Abstract

Additive manufacturing (AM) of dense pastes is attracting interest across many fields and while the combination of a high loading of functional particles with a flowable continuous phase is promising, the heterogeneous system makes material and process design difficult. Prior work has focused on assessing printability by examining their extrudability and shape retention. However, for dense pastes, the inks are susceptible to separation of particles from binder, so we consider composition consistency as a critical quality attribute. This composition consistency attribute provides a framework for quality assessment of material extrusion AM inks and processes.

**Graphical abstract:**

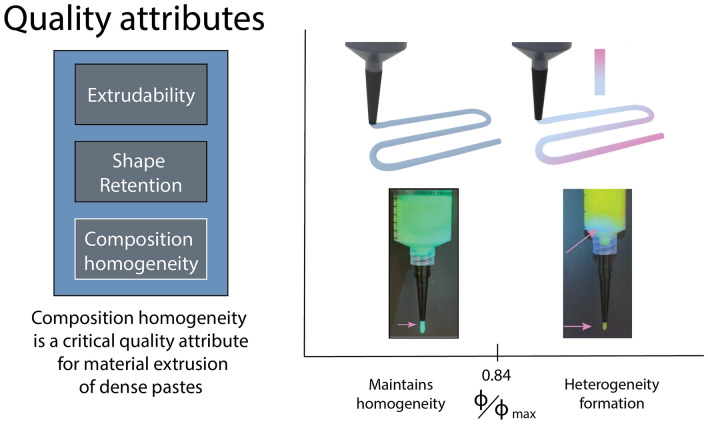

**Supplementary Information:**

The online version contains supplementary material available at 10.1557/s43579-026-00936-9.

## Introduction

Additive manufacturing (AM) has become increasingly appealing due to its ability to rapidly and precisely fabricate complex structures. AM offers design flexibility with customizable geometries, produces less waste due to material efficiency, and often reduces overall cost and production time compared to traditional expensive machinery.^[1]^ As one branch of material extrusion AM, direct ink write (DIW) deposits a continuous flow of an ink with controlled rheological properties layer-by-layer onto a substrate.^[2]^ DIW inks often consist of particles suspended in a polymer matrix. These inks are highly customizable, including particles such as organic biogels,^[3]^ conductive metals,^[4]^ lunar regolith,^[5]^ or electrically or thermally insulating ceramics^[6]^ suspended into a polymer matrix or similar base. The polymers selected include epoxies,^[4]^ UV-curable acrylates,^[5]^ high molar mass polymers dissolved in a solvent,^[7]^ or conductive polymers like polypyrrole.^[8]^ Because of the wide-ranging material space available, DIW is seeing implementation in electronics,^[4,8]^ medical devices,^[3]^ aerospace exploration^[5]^, among other fields.

For certain applications such as energetics^[4]^ and ceramics,^[6]^ the composite materials require a high density of particle content, or solids loading, for successful implementation.^[2]^ These high-solid composites are desirable to improve shape retention—the ability to hold its printed shape^[9]^—by minimizing shrinkage post-extrusion. Poor shape retention is most common in low-solids formulations and may be defined by slumping^[10]^ or line width fluctuations.^[11]^ Conversely, as the solids loading increases beyond 50 vol%, the dense pastes can impede extrusion due to rapid increases in viscosity caused by particle–particle interference.^[12]^ Specific defects seen previously include void formations caused by air bubbles, tail effects, or interfilament voids.^[13]^ At higher solids content or stronger extrusion forces, prints may be obstructed by nozzle jamming^[14]^ or crack propagation.^[15]^

Assessing the printability of a new ink is essential to developing quality parts and “printability” is typically defined as the ability of an ink to be extruded and hold its shape. Without smooth, constant flow or maintaining the part’s geometry after being deposited, DIW loses the benefits that make it an attractive choice over traditional manufacturing methods. Therefore, understanding the printability of an ink, especially in problematic high-solids regimes, is extremely important when designing formulations and processing parameters. Researchers have been increasingly interested in linking measurable formulation and processing parameters to the printability of inks to decrease trial-and-error work. The material properties, including solids loading, particle size distribution, and binder viscosity and molecular weight, as well as the processing parameters, which include extrusion pressure, print head speed, and the mechanism of solidification, impact the print’s quality, shape fidelity, and mechanical reliability.^[1,16]^ Existing studies have linked axial stress,^[9]^ yield stress,^[10]^ and storage modulus^[17]^ as important material parameters to an ink’s printability.

One challenge in previous efforts exploring these relations is the inconsistent or incomplete definition of printability. Most works adopt the final shape retention as the most important factor;^[10,11]^ others consider rheological parameters, such as storage modulus or yield stress, as quantitative proxies for extrudability.^[9]^ Especially lacking are printability studies focusing on dense pastes with high solids content. Griebler et al. characterized printability for inks between 40 and 74 vol% of various fillers, reaching high solids regimes.^[9,17]^ They used a precisely defined set of printability attributes: (1) ink must be extrudable with a nozzle diameter of 0.84 mm and flow rate of 0.25 mL/min (extrudability criterion) and (2) height of a printed cone must not change by more than 10% in one minute (shape retention criterion). They related printability to rheological characteristics previously shown to be important as well as a parameter for how close the solid filler loading is to the theoretical maximum packing fraction, $\phi /{\phi }_{max}$.^[9,18]^ They found that for the defined extrudability and shape retention attributes, only $\phi /{\phi }_{max}$ clearly predicted printability of inks for a wide range of particles in a silicone binder.

Although the extrudability and shape retention attributes are important for quality of printed materials, for DIW of high solids materials, another attribute may be equally important to assess: the homogeneity of the composition from start to end of the print. At high solids contents, particle rearrangement is common in response to applied shear, gravity or other forces.^[19]^ One frequently noted aspect of this relevant to 3D printing is the formation of a slip layer and particle mat^[14,20]^ during extrusion. The radial gradient of shear rates during extrusion in a cylindrical or conical geometry has been observed to alter the composition of DIW composite inks, with binder-rich regions forming in high shear zones at the extruder wall, and particle-rich regions forming along the centerline.^[20,21]^ Other composition change phenomena such as settling and aggregate formation^[12]^ can still be appreciable at moderate solid loadings and/or highly mismatched bimodal particle size distributions.^[22]^ Printed parts are typically designed with the assumption that smooth extrusion constitutes consistent ink composition throughout the print, but it is crucial to assess this property when developing quality plans for manufacturing via DIW of these complex inks.

In this work, we define a new metric for “printability” based on the formation of heterogeneities during the printing process and examine whether that metric is affected by potential critical material properties ($\phi /{\phi }_{max},$ vol% coarse particles, binder type) and critical process parameters (nozzle diameter and decrease in diameter from barrel to nozzle). This strategy aligns with principles of “quality-by-design” (QbD), where critical quality attributes (CQA, “printability metrics” in the additive manufacturing community) are first identified and then the material properties and process parameters that may affect them are assessed to identify the most important for quality control.^[23]^ Here, we quantify the particle-binder migration for 15 different formulations to test our quality metric for a wide range of materials, using model glass particles with bimodal particle size distributions and polymer binder systems that span a range of properties and include UV curing monomers, UV curing oligomers, and solvent-evaporating polymer/solvent binder systems. This approach identifies $\phi /{\phi }_{max}$ as a critical material property, consistent with prior work by Griebler et al.^[9,17]^ but for a newly defined CQA. The CQA is defined as maintaining a consistent composition from the start to the end of a print (< 10% change in composition) that we show is distinct from the “extrudability” and “shape retention” attributes defined previously. This work provides a framework to more completely assess quality of prints from high solids inks, which are sensitive to heterogeneity formation during flow and thus require consideration of this attribute.

## Materials and methods

### Ink formulation

Fifteen inks were prepared containing > 50 vol% glass microspheres suspended in various binders and the formulations are described in Table I. The glass microspheres were sourced from Potters Industries and included two distinct sizes: the coarse particles (A-1820) had a d_10_ = 235 μm, d_50_ = 271 μm, and d_90_ = 315 μm, and the fine particles (A-3000) had a d_10_ = 10 μm, d_50_ = 35 μm, and d_90_ = 80 μm, as measured via light scattering on a Malvern Mastersizer 2000SM. Full distributions are shown in Supplementary Figure S-1, and SEM images portraying sphericity are shown in Supplementary Figure S-2. The monomers and oligomers used for the UV cure binders were sourced from Sigma Aldrich and included poly(ethylene glycol) diacrylate (PEGDA, Mw 700), 2-ethylhexyl acrylate (EHA), and triethylene glycol dimethacrylate (TEGDMA). The photoinitiator used was 1 wt% Irgacure-819 sourced from Aldrich. The polyurethane diacrylate resin Ebecryl 230 was provided by Allnex. The polymer used to examine polymer/solvent systems was polystyrene-block-polyisoprene-block-polystyrene (SIS), obtained from Sigma Aldrich and this was dissolved in toluene, sourced from VWR. The inks were produced in batches of 10 mL and were mixed in a Flacktek 400.2VAC-L dual-axis centrifugal mixer (DAC) for 1 min at 1000 rpm, followed by 45 s at 0 rpm, and 45 s at 1500 rpm. These conditions were demonstrated in prior work to result in well-mixed pastes.^[5]^ The formulations consist of nine inks with assorted UV cure binders and six inks with polymer/solvent systems. The formulations consist of solids loading from 54 to 65 vol%, and with distinct coarse to fine ratios of 65:35, 71:29, 77:23, and 100:0. The formulation IDs and their respective compositions are tabulated in Table I.

**Table I:** Composition of the fifteen inks evaluated.

ID #	Binder composition (vol:vol)	Solid loading (vol% of total formulation)	Coarse particles (vol% of total particle composition)	Fine particles (vol% of total particle composition)
ID1	1:1 PEGDA:EHA	65	71	29
ID2	2:1 PEGDA:EHA	65	71	29
ID3	1:1 PEGDA:EHA	65	65	35
ID4	2:1 PEGDA:EHA	65	65	35
ID5	1:10 PEGDA:EHA	65	65	35
ID6	1:1 PEGDA:EHA	60	100	0
ID7	TEGDMA	60	71	29
ID8	Ebecryl 230	50	71	29
ID9	1:1 PEGDA:EHA	65	77	23
ID10	25% SIS	65	71	29
ID11	15% SIS	65	71	29
ID12	15% SIS	62	65	35
ID13	25% SIS	62	65	35
ID14	25% SIS	54	71	29
ID15	25% SIS	58	71	29

PEGDA: poly(ethylene glycol) diacrylate, EHA: 2-ethylhexyl acrylate, TEGDMA: triethylene glycol dimethacrylate, Ebecryl 230: a commercial polyurethane diacrylate, SIS: polystyrene-block-polyisoprene-block-polystyrene dissolved at the noted wt% in toluene. Particles are glass microspheres from Potters Inc. Coarse particles d_50_ = 270 μm, fine particles d_50_ = 35 μm.

### Rheology

A TA Instruments DHR-3 rheometer equipped with 25 mm crosshatched parallel plates was used to conduct shear rate sweeps of the fifteen inks. The inks were loaded onto the rheometer plates and a gap height of 1000 μm was used. A preshear step was applied first to remove any stress history on the materials and consisted of 150 s of oscillation at 5 Pa and 10 rad/s followed by 150 s at 1 Pa and 10 rad/s. After the preshear step, a flow sweep from 0.01 s^−1^ to 500 s^−1^ was conducted. The temperature was kept constant at 25℃ during all tests, and a solvent trap was used to protect any exposed ink from ambient light and to limit solvent evaporation where applicable. Three individual samples were run for each of the fifteen inks in this manner, and the viscosity curves obtained were averaged.

To measure the zero-shear viscosity of the binders only (Supplementary Figure S-3), the DHR-3 rheometer was equipped with a double-gap concentric cylinder geometry and loaded with 10 mL of the polymer binders. A flow sweep with shear rates from 0.01 s^−1^ to 500 s^−1^ was conducted at constant temperature of 25℃ with a solvent trap. The linear plateau region was extracted, and a zero-shear viscosity value was extrapolated as the shear rate approached zero. Three individual samples were run for each of the polymer binders, and the viscosity curves obtained were averaged.

### Extrusion nozzle design

To evaluate the effect of process parameters related to the nozzle on quality, three different printer syringes and nozzles were compared. The selected syringe barrel and nozzle pairs are combination A (10 mL syringe, 14G nozzle), combination B (20 mL syringe, 14G nozzle), and combination C (60 mL syringe, 10G nozzle). The two nozzle sizes were chosen to be 5–10 × larger than the larger particle size (d_50_ of 270 μm), per standard practice in composite ink 3D printing literature.^[24]^ Regardless of nozzle or barrel size, printing was performed with an ink volume of 10 mL. The three combinations were selected to provide two points of comparison: A and C show the effect of estimated shear rate in the nozzle, and A and B or B and C show the effect of the ratio of shear rate in the barrel to that in the nozzle. A general approximation for the wall shear rate during printing was made using Eq. 1, where the wall shear rate ${\dot{\gamma }}_{wall}$ is a function of both the ink flow rate ($Q$) and the extruder geometry (radius, $R$).^[25]^ Since the formulations all fall within similar particle loadings and are tested in a small range of flow rates, we use the Newtonian approximation for an estimated comparison between systems even though these are non-Newtonian pastes. For the nozzle, we only consider the estimated shear rate at the nozzle exit (the nozzle is conical) for comparison of different extruder assemblies.

1 $${\dot{\gamma }}_{wall}=\frac{4Q}{\pi {R}^{3}}$$

The nozzle inner diameters were 1600 μm for the 14G nozzle and 3000 μm for the 10G nozzle. The layer height was programmed to be 1000 μm, which, at 0.625 × the nozzle diameter, follows previous DIW guidelines to support layer interconnectivity.^[26,27]^ For each barrel and nozzle setup, a reasonable $Q$ was selected and programmed to enable smooth, consistent flow. For the 10 mL/14G and 20 mL/14G configurations, $Q$ was 16 mm^3^/s. For the 60 mL/10G configuration, $Q$ was 56 mm^3^/s. In this manner, the shear rates in the barrel and nozzle were calculated for each syringe barrel and nozzle combination, as shown in Supplementary Table T1. Briefly, combination A (10 mL syringe with 14G nozzle) was estimated to have a shear rate of 0.05 s^−1^ in the barrel and 40 s^−1^ in the nozzle, combination B (20 mL syringe with 14G nozzle) was estimated to have a shear rate of 0.02 s^−1^ in the barrel and 40 s^−1^ in the nozzle and combination C (60 mL syringe and 10G nozzle) was estimated to have a shear rate of 0.03 s^−1^ in the barrel and 21 s^−1^ in the nozzle.

### Printing and assessment of composition change

Each of the fifteen inks were extruded with the three setups by use of a syringe pump oriented vertically (to mimic DIW extruders). 10 mL aliquots of inks were loaded and the corresponding barrel diameter, ink volume, and selected flow rates were programmed into the syringe pump for each syringe geometry. Extrusion quality was observed visually and samples for a quantitative analysis were collected on small 20 mm x 25 mm alumina ceramic sheets after 1 mL of ink was extruded (start of extrusion) and another was collected when less than 2 mL of ink remained in the barrel (end of extrusion). Note that not all samples extruded fully from the syringes, in which case the plunger was removed, and a small amount of ink was directly retrieved from the barrel and placed on the alumina sheet. The samples were cured or dried and weighed, then placed in a Thermolyne benchtop muffle furnace in an air environment at 600℃ for 1 h to burn out the binder. After cooling, each alumina sheet was carefully removed from the furnace and the new sample mass was recorded to measure the mass of binder lost after the organics burn out, as calculated with Eq. 2.

2 $${\mathrm{Mass}}\% \,{\mathrm{binder}}\, = \,\frac{{{\mathrm{Mass}}\,{\mathrm{prior}}\,{\mathrm{to}}\,{\mathrm{furance}}\, - \,{\mathrm{mass}}\,{\mathrm{remaining}}\,{\mathrm{after}}\,600^{^\circ } {\mathrm{C}}\,{\mathrm{for}}\,1{\mathrm{h}}}}{{{\mathrm{Mass}}\,{\mathrm{prior}}\,{\mathrm{to}}\,{\mathrm{furnace}}}}$$

The change in composition was quantified by:

3 $$\% {\mathrm{composition}}\,{\mathrm{change}}\, = \,\frac{{{\mathrm{binder}}\,{\mathrm{at}}\,{\mathrm{end}}\,\left( {{\mathrm{mass}}\% } \right)\, - \,{\mathrm{binder}}\,{\mathrm{at}}\,{\mathrm{start}}\,({\mathrm{mass}}\% )}}{{{\mathrm{binder}}\,{\mathrm{at}}\,{\mathrm{start}}\,({\mathrm{mass}}\% )}}$$

Equation 3 quantifies the change in composition of the extruded inks from the start to end of extrusion. We have defined a minor composition change to have occurred if the quantity of binder has changed by > 5% and a significant composition change with > 10%. Three repeat samples at both the start of extrusion and end of extrusion were collected for each ink in each extruder setup.

### Microcomputed tomography (μCT)

Cross-sectional imaging of printed lines was performed on a Scanco Medical μCT50. Straight ink lines were extruded from a Hyrel Hydra 21 3D printer installed with an EMO-25 cold flow extruder DIW head onto a glass substrate. Formulations with UV-curing polymer binders were cured during the print under 365 nm UV radiation while solvent evaporation systems were left to dry for 3 h at room temperature. These lines were scanned in the μCT50 at a resolution of 3 μm.

## Results and discussion

When printing inks with high particle loadings, there are many ways in which quality of the print is insufficient for applications. Most studies have focused on the ability to extrude smoothly^[10,13]^ or the ability to build new layers on top of existing ones^[10]^ and have even developed generalized design rules for these quality parameters.^[1,9,16]^ However, high solids inks are particularly vulnerable to formation of heterogeneities within an extruded filament through migration of particles or binder.^[21]^ Here we focus on the formation of sufficiently large heterogeneities that the composition, as characterized by the binder to particle ratio, changes from the start of the end of the print. Such changes lead to changing material properties from start to end of the print and thus are clear indicators of poor print quality.

**Figure 1:**
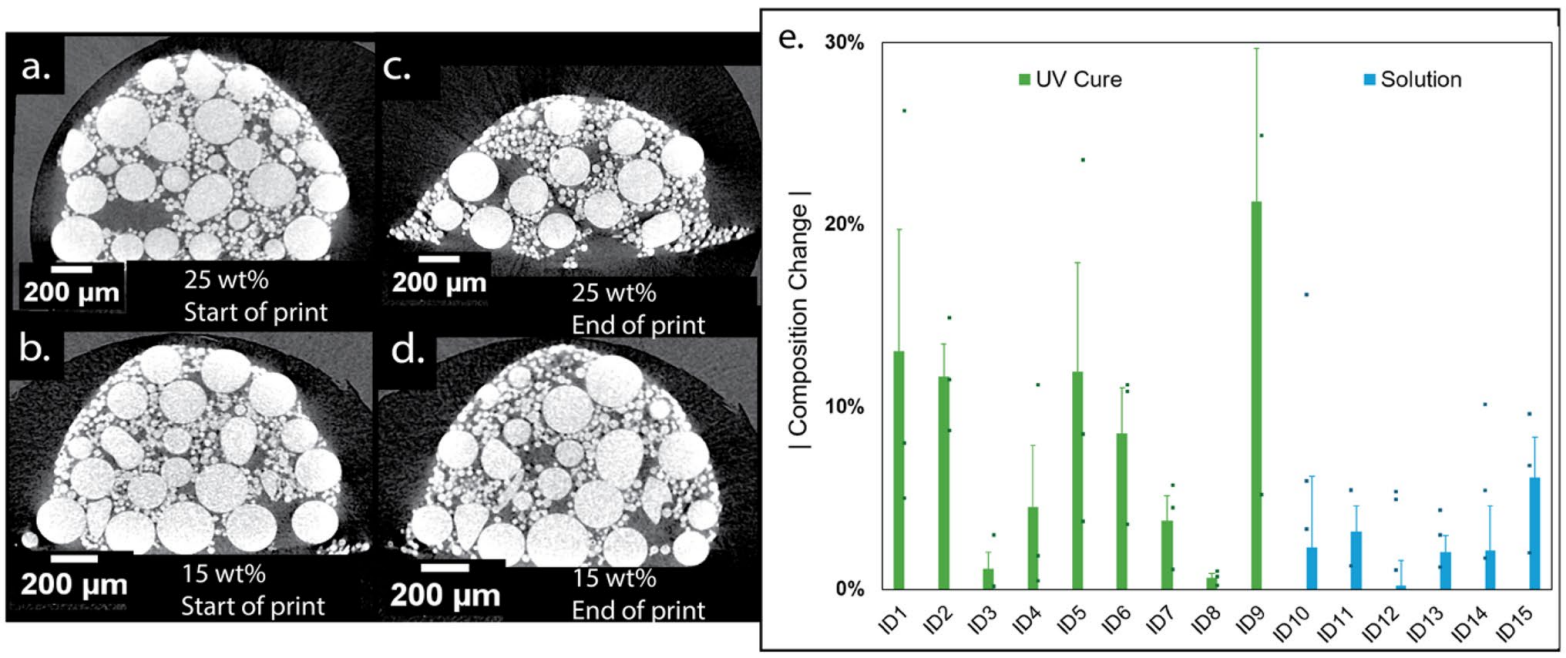
μCT images of 65 vol% solids formulations with 71:29 coarse: fine bimodal particle size distributions and (a) 25 wt% SIS in toluene binder at the start of the print, (b) 15 wt% SIS in toluene binder at the start of the print, (c) 25 wt% SIS in toluene binder at the end of the print, (d) 15 wt% SIS in toluene binder at the end of the print, and (e) absolute value of the composition change for all formulations tested. Individual data points plotted as well as the mean and standard deviation.

Figure 1 shows μCT images of lines printed from two formulations containing 65 vol% particles with a 71:29 volume ratio of coarse to fine particle sizes, one with a binder composed of 25 wt% SIS in toluene (a and c) and the other with 15 wt% SIS in toluene (b and d). The 25 vol% SIS in toluene formulation shows appreciable differences in binder to particle ratio from the start to the end of the print when printed with this nozzle configuration (decrease in binder mass% of 16.16 ± 4.29%), while the 15 wt% SIS in toluene does not (1.31 ± 4.21%). From the μCT we can see that the shape of the printed filament also changes for the 25 wt% SIS in toluene, but it does not change for the 15 wt% SIS in toluene binder formulation, which is likely due to “slumping” after printing that may be more severe in at the end of the print due to the compositional changes. This is one indicator of the effect of composition change on print quality.

Although analysis of particle vs. binder areas in μCT images can give some indication of heterogeneity formation, quantification is difficult due to low contrast between the binder and the background, the small size of fine particles and cost of obtaining sufficient sample sizes for statistical analysis. Thus, we instead use a gravimetric method to quantify the change in binder: particle ratio from the start to the end of the print, quantifying this change as the % change in binder mass (Eq. 3). The absolute value of the percent change for all formulations is shown in Fig. 1(e). The initial composition of the fifteen inks evaluated may be reviewed in Table I. The average composition changes range from nearly 0% to 20%, with six formulations showing appreciable average composition changes > 5% and four showing changes > 10%. Small fluctuations in composition change are anticipated due to the formation of a slip layer or particle mat in dense pastes during extrusion. However, significant composition change over the 10% composition change threshold indicates a detrimental heterogeneity formation despite apparent extrudability. The results in Fig. 1 show that for a variety of inks (shear rate sweeps for these inks shown in supplementary information Figure S-4, with zero-shear viscosities ranging from $1.0\times {10}^{3}-3.0\times {10}^{5} Pa\cdot s$), the heterogeneity formation is sufficiently high to impact the composition as the extrusion progresses despite the continued ability to extrude the inks. This highlights composition consistency as an important quality parameter for DIW printing of highly loaded inks.

**Figure 2:**
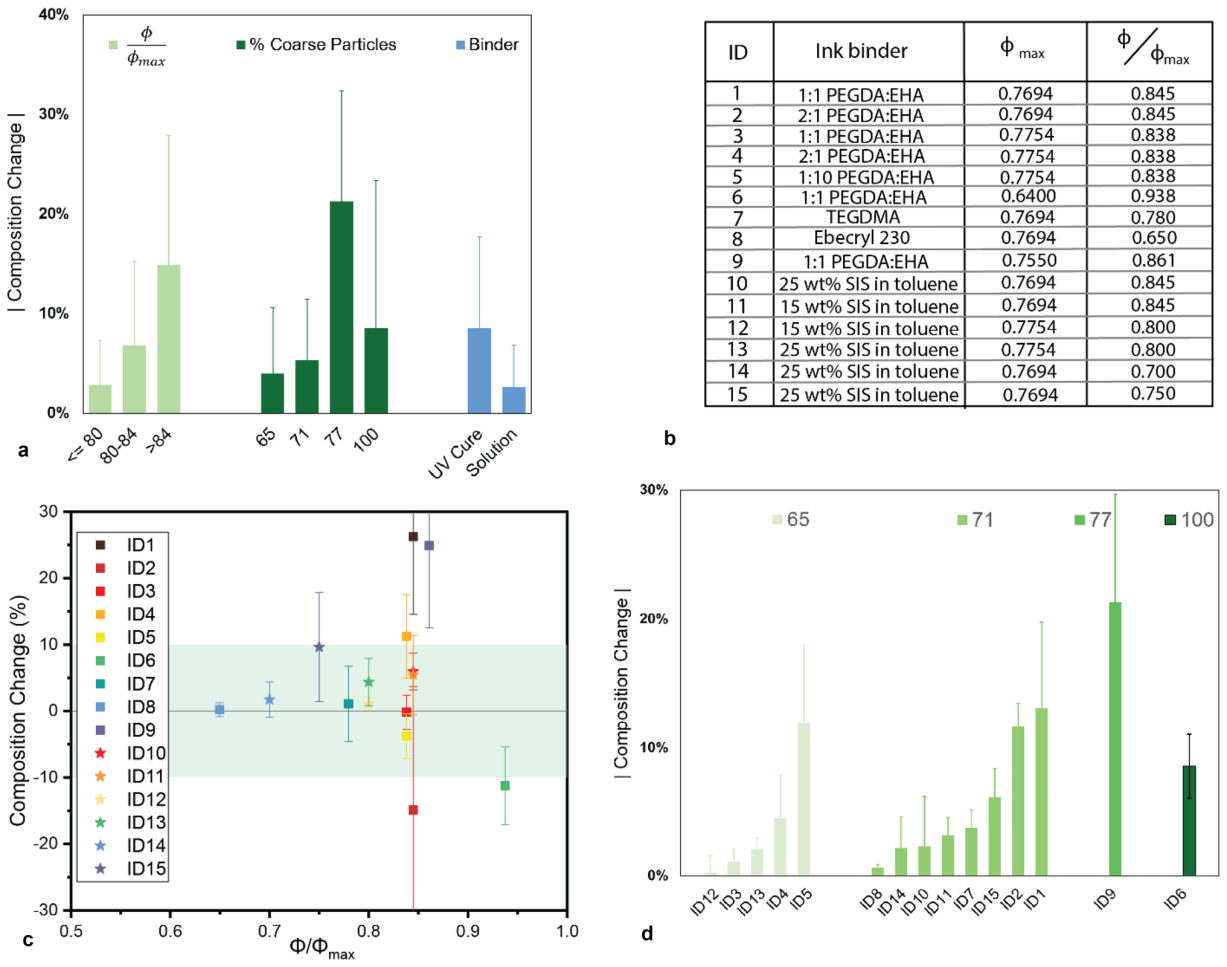
(a) Absolute value of averaged composition change data for different groups of material properties. Error bars are standard deviation of all samples and replicates in that group, (b) table containing the $\frac{\phi }{{\phi }_{max}}$ values for all formulations, (c) averaged composition change data for all formulations as a function of the calculated particle packing ratio, $\frac{\phi }{{\phi }_{max}}$, shaded green region designates composition changes < 10% (d) absolute value of average composition change for increasing ratios of coarse particles in the bimodal combinations of glass fillers ranging from 65 to 100%. Results are grouped by vol% coarse particles (light green = 65 vol% moderate green = 71 vol%, dark green = 77 vol%, and blue = 100 vol%).

In the QbD framework, once a CQA is identified, it is important to assess the impact of formulation and processing parameters on that quality parameter. Here we hypothesize that formulation properties previously identified to impact extrudability and shape retention will also impact heterogeneity formation as measured through composition change. We specifically focus on the ratio of the packing fraction to the theoretical maximum packing fraction ($\frac{\phi }{{\phi }_{max}}$) and the vol% coarse particles relative to all particles. As we are also testing both UV cure and polymer solution-based binders, we will also examine binder type. Figure 2(a) shows the absolute value of the average % composition change across all formulations and nozzle geometries tested with results grouped for these parameters.

As the loading of particles increases in a formulation, it reaches a point of greatest particle density where a continuously printed material is still achievable. It is known that the closer an ink is to its maximum packing fraction, the more difficult it is to extrude due to jamming effects or crack propagation,^[12,14,15]^ and Griebler et al. showed that inks with $\frac{\phi }{{\phi }_{max}}>0.94$ led to behavior classified as “jammed.” We expect that heterogeneity formation will also become more severe as we approach ${\phi }_{max}$. To assess this, a theoretical ${\phi }_{max}$ was calculated following Eq. 4 to determine where the maximum packing fraction lies at each of the bimodal ratios. This model, derived by Zheng, Carlson, and Reed, expands on the Furnas model and calculates the theoretical maximum packing fraction of a bimodal system based on the coarse:fine particle size ratio, the packing efficiency, and the volume fraction of the powders.^[28]^

4 $${\phi }_{max}={PE}_{c}+\left(1-{PE}_{c}\right)*{PE}_{f}*{\left[\mathrm{e}*{x}_{f}ln\left({x}_{f}\right)\right]}^{\frac{5}{4{PE}_{c}}}*exp \bigg(\frac{-4{R}_{f}}{{R}_{c}} \bigg)$$

where ${PE}_{c}$ and ${PE}_{f}$ are the monomodal packing efficiency of coarse and fine particles, respectively, and are assumed to be 0.64 since the particles are spherical (images in Supplementary Figure S-2).^[^^18,29^^]^ ${R}_{c}$ and ${R}_{f}$ are the radii of the coarse and fine particles, in this case using the d_50_ value, *e* is the base of a natural logarithm, and ${x}_{f}$ is the fraction of fine particles. From Eq. 4, the theoretical ${\phi }_{max}$ of the three distinct volume ratios of coarse to fine particles—65:35, 71:29, and 77:23—were computed to be 0.775, 0.769, and 0.755, respectively. We note that Griebler et al.^[9]^ used a rheological method to determine ${\phi }_{max}$ for a variety of particle inks rather than a theoretical estimate. We did not use this approach because our study uses only glass spheres and thus any error in the estimate from Eq. 4 would result in a systematic error and not change general trends.

For each formulation, we calculate $\frac{\phi }{{\phi }_{max}}$, a parameter that quantifies how close the packing fraction is to the theoretical maximum packing fraction and these values are shown in Fig. 2(b). Figure 2(c) shows the average composition change and standard deviation for all formulations plotted as a function of the $\frac{\phi }{{\phi }_{max}}$. The composition change remains minor at low values of $\frac{\phi }{{\phi }_{max}}$, but rapidly becomes more variable as $\frac{\phi }{{\phi }_{max}}$ reaches 0.84. This indicates that there is a threshold packing where heterogeneity formation begins. Interestingly, Griebler et al. found that the “jammed” inks occurred at $\frac{\phi }{{\phi }_{max}}>0.94$ and that “runny” inks, which did not maintain their shape after printing, occurred at $\frac{\phi }{{\phi }_{max}}<0.9$. This discrepancy may be due to differences in determining ${\phi }_{max}$ or it may be an indicator that the printability criterion of maintaining compositional homogeneity fails at a lower $\frac{\phi }{{\phi }_{max}}$ than Griebler et al.’s extrudability criterion.

One distinction between the materials studied in this work and Griebler et al. is their use of solely formulations that did not cure (silicone-based) and thus the need to maintain their shape after printing relies solely on the mechanical stability of the ink.^[17]^ In our case, we use UV-cure binders, which are cured immediately after extrusion, and solvent-evaporation binders (toluene, BP ~ 111℃), which are slower to solidify than UV, but experience surface solidification on the order of seconds. Thus, we cannot compare the lower bound of Griebler et al.’s range, defined as “runny,” to our formulations and there is no inherent conflict between our printability criterion of $\frac{\phi }{{\phi }_{max}}<0.84$ to avoid heterogeneity formation and their lower bound. However, this does raise a question of whether the polymer binder type is tied to whether the formulation tends to experience heterogeneity formation. Based on the materials tested here (UV cure—acrylates, solvent evaporation—SIS in toluene), the UV cure formulations have a greater tendency to change composition from the start to the end of the print [Fig. 2(a)]. This may be due to stabilizing effects of higher molar mass polymers;^[2]^ the polymer/solvent SIS formulations showed a higher average zero-shear viscosity compared to the UV cure formulations, which may be seen in the shear rate sweeps in Supplementary Figure S-4. However, a greater variety of binder systems must be tested to confirm this relationship, which is outside the scope of this work.

Based on prior knowledge of the field, we hypothesized and demonstrated that $\frac{\phi }{{\phi }_{max}}$ would be a critical formulation parameter affecting heterogeneity formation. However, we also examined the vol% of coarse particles independent of $\frac{\phi }{{\phi }_{max}}$ as well as process parameters based on the nozzle configuration. We selected vol% coarse particles because we expect that fine particles will move more readily and may experience migration during extrusion similar to the binder layer. However, Fig. 2(d) shows that there is no clear trend with the vol% coarse particles—some formulations in each category show high variability in composition change, while others do not. Hence, we can rule out vol% coarse particles as a critical parameter applicable across varied formulations (however, it could still be relevant in more narrow material groups).

**Figure 3:**
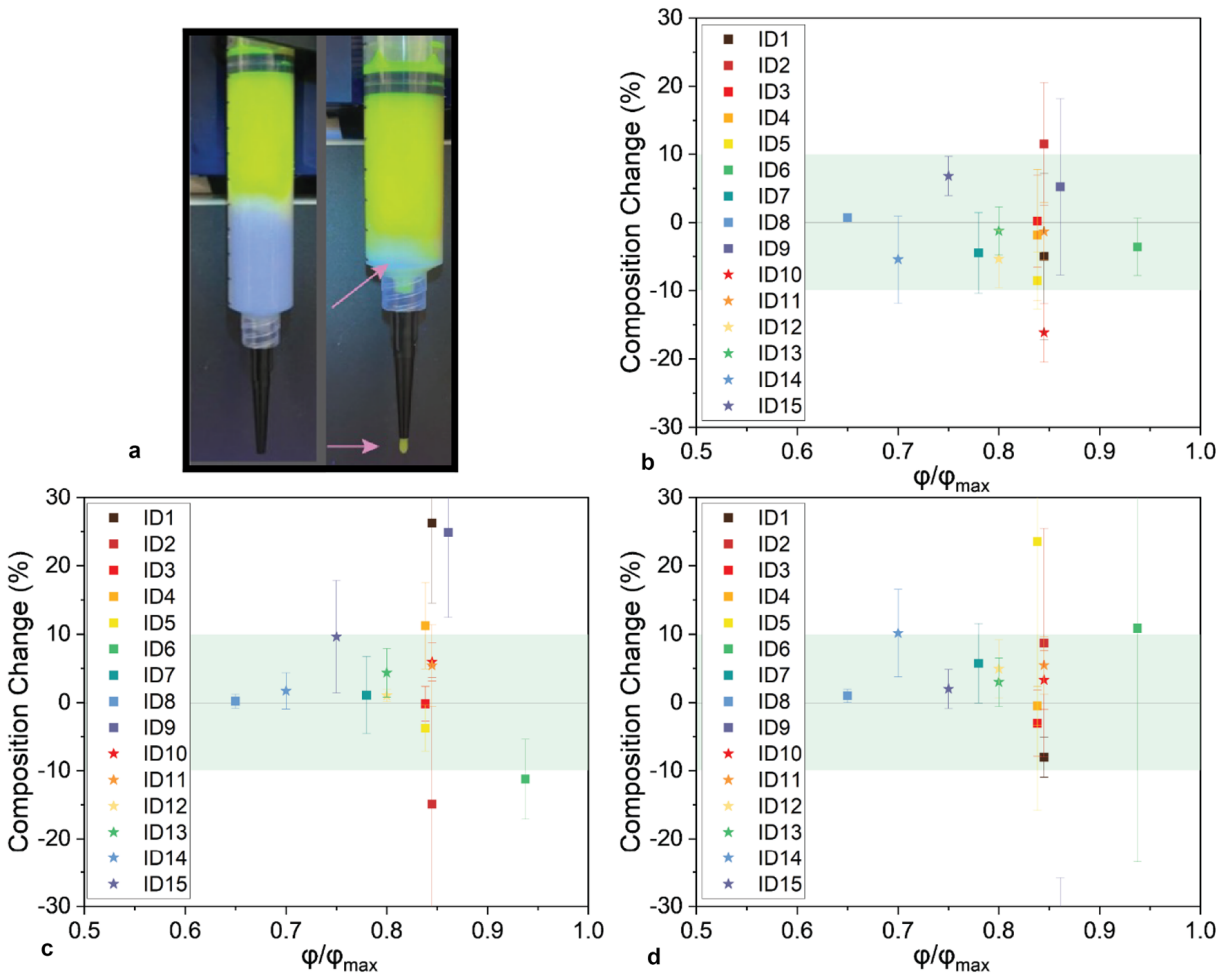
(a) Snapshot during the extrusion of ID1 in the 10 mL, 14G setup showing dead zones with segregated inks. ID1 consists of 1:1 PEGDA:EHA with 65% solids loading of 71:29 coarse: fine particles. (b) Measured composition change with respect to the calculated $\frac{\phi }{{\phi }_{max}}$ particle packing, based on extrusion through a 10 mL/14G sample, (c) based on extrusion through a 20 mL/14G samples, and (d) based on extrusion through a 60 mL/10G samples. Shaded regions in b-d indicate composition changes < 10%.

We selected the nozzle design as a potential critical processing parameter for heterogeneity formation because, for a given flow rate, the shear rate experienced in the nozzle will vary with its diameter, seen in Eq. 1. Additionally, we anticipated that sharp changes in the barrel and nozzle diameters could destabilize flow and lead to greater heterogeneity formation. This can be seen in Fig. 3(a), where a dye-free formulation was inserted into the syringe first, followed the same formulation containing a small amount of fluorescent dye. When the boundary between the clear and dyed segments reached the transition point from the syringe barrel to the nozzle, we visualized radial flow variations. Specifically, a portion of the dye-free portion was still visible in the syringe barrel while dyed ink was extruding from the nozzle.

We tested 3 nozzle configurations (Supplementary Table T1), with combination A and C designed to assess the effect of shear rate in the nozzle alone and combination A and B designed to assess the effect of the change in shear rate from the barrel to nozzle. As can be seen in Fig. 3(b)–(d), when the data is taken together, there are no clear ties between nozzle design and composition change independent of the role of $\frac{\phi }{{\phi }_{max}}$. Despite the radial flow variations in the barrel, the shear stresses applied on the inks by the walls change insignificantly between geometry configurations. Recall, our previous approximations of the barrel shear rates were 0.05 s^−1^, 0.02 s^−1^, and 0.03 s^−1^ for a barrel volume of 10 mL, 20 mL, and 60 mL, respectively, and the nozzle shear rates were 40 s^−1^ and 21 s^−1^ for 1600 μm and 3000 μm. The change in viscosity at these shear rates, even in shear thinning formulations, is minimal. As a result, the transition to inhomogeneous flow occurs at $\frac{\phi }{{\phi }_{max}}>0.84$ for all geometries.

## Conclusion

Increasing the pace of product and process development requires the ability to predict the effect of formulation and process parameters on the quality of the product. Thus, to develop design rules it is important to identify the critical quality attributes (CQAs). Prior efforts have focused on the extrudability of the ink and shape retention, but here we show that for dense pastes, the ability to maintain composition from start to end of a print can also be a CQA. This is particularly expected in dense pastes due to known phenomena such as formation of a slip layer and particle mat that occur due to complex interactions of particles with each other, the binder and the nozzle during flow. We show that composition changes occur in many formulations that can be extruded and that this can be quantified through measurement of the binder to particle ratio at the start and end of the print. Although the focus of this study was not to identify the relationship between all material and process parameters and the new CQA, we examined 15 formulations and 2 process parameters and determined that the ratio of the packing fraction to the theoretical maximum packing fraction ($\frac{\phi }{{\phi }_{max}}$) has a significant impact on the composition consistency CQA. This adds support for $\frac{\phi }{{\phi }_{max}}$ as a critical material parameter for material extrusion additive manufacturing, as it has previously been shown to be crucial for extrudability and shape retention.^[9,17]^ Future work assessing the impact of other material and process parameters on composition consistency will enable development of design rules to print quality parts from dense pastes for applications ranging from ceramics to energetic materials to pharmaceuticals.

## Supplementary Information

Below is the link to the electronic supplementary material.

Supplementary file1 (DOCX 900 KB)

## Data Availability

Data for this manuscript is provided in the manuscript or in the supplementary information.
