# Supplementary material for: Composition consistency as a critical quality attribute for material extrusion additive manufacturing
Source: MRS Commun. 2026 Feb 17;16(3):566–74. doi: 10.1557/s43579-026-00936-9 (PMC13337734; doi:10.1557/s43579-026-00936-9)
Supplement: Supplementary file 1 — Supplementary file1 (DOCX 900 KB) [file 43579_2026_936_MOESM1_ESM.docx]

**Supplementary Information**

# **Composition consistency as a critical quality attribute for material extrusion additive manufacturing**

Laurel Hilger^1 ∥^, Alexandra Marnot^2 ∥^, Blair Brettmann^1,2 *^

^1^Georgia Institute of Technology, School of Materials Science and Engineering, Atlanta, GA 30332, USA

^2^Georgia Institute of Technology, School of Chemical and Biomolecular Engineering, Atlanta, GA 30332, USA

*Corresponding author: Blair Brettmann, blair.brettmann@chbe.gatech.edu

^∥^Authors contributed equally

## S1. Particle Size Distributions of Solid Particles


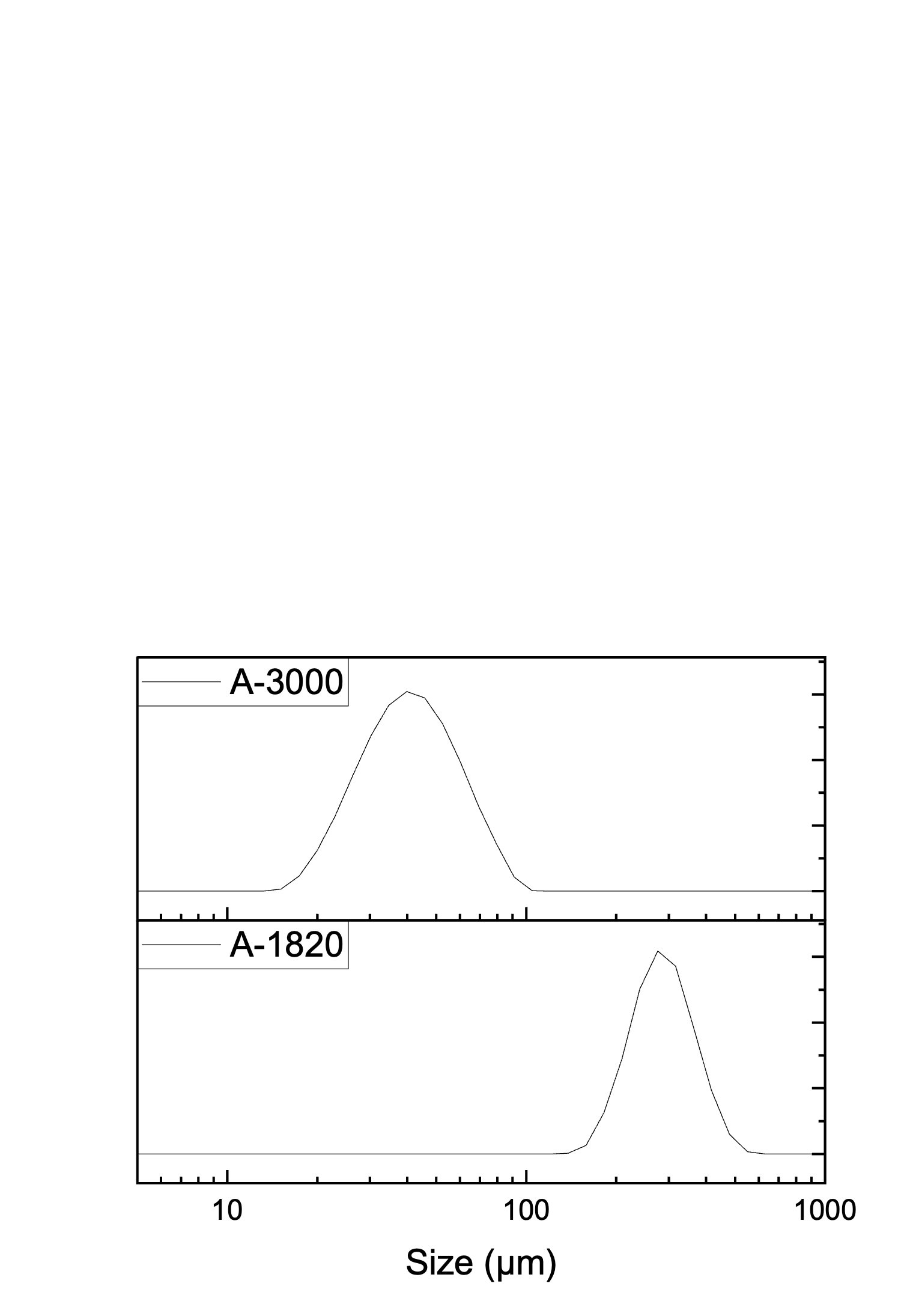


**Figure S1: Particle size distributions of Potters Spheriglass beads. Fine particles were Potters A-3000 with a d_50_ =** $\boldsymbol{41}\boldsymbol{\mu m}$**and a dispersity** $\boldsymbol{\sigma}^{\boldsymbol{2}}\boldsymbol{=0.130}$**. Coarse particles were Potters A-1820 with a d_50_ =** $\boldsymbol{283}\boldsymbol{\mu m}$**and a dispersity** $\boldsymbol{\sigma}^{\boldsymbol{2}}\boldsymbol{=0.056}$

## S2. SEM Images of Solid Particles


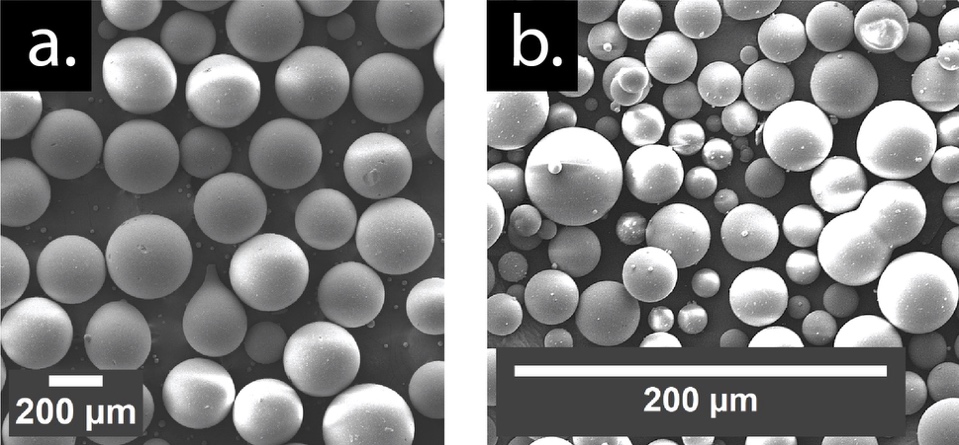


**Figure S2: SEM images of a) coarse and b) fine solid particles used in formulations to view sphericity. Coarse particles have an aspect ratio of 1.049. Fine particles have an aspect ratio of 1.058.**

**T1:** **Selected plastic syringe volumes and nozzle gauges for assessment of nozzle-geometry process parameters. Shear rates are apparent wall shear rates computed according to Equation 1**

| **Combo** | **Syringe barrel**  **(inner diameter)** | **Shear rate,** $\gamma_{barrel}$**, (s^-1^)** | **Nozzle gauge (inner diameter)** | **Shear rate,** $\gamma_{nozzle}$**, (s^-1^)** | **Shear rate ratio,** ${\gamma_{barrel}}/{\gamma_{nozzle}}$ |
| --- | --- | --- | --- | --- | --- |
| A | 10 mL (14.5 mm) | 0.05 | 14G (1.6 mm) | 40 | 0.0013 |
| B | 20 mL (19.13 mm) | 0.02 | 14G (1.6 mm) | 40 | 0.00059 |
| C | 60 mL (26.7 mm) | 0.03 | 10G (3 mm) | 21 | 0.0014 |

## S3. Zero-Shear Viscosities of Polymer Binders


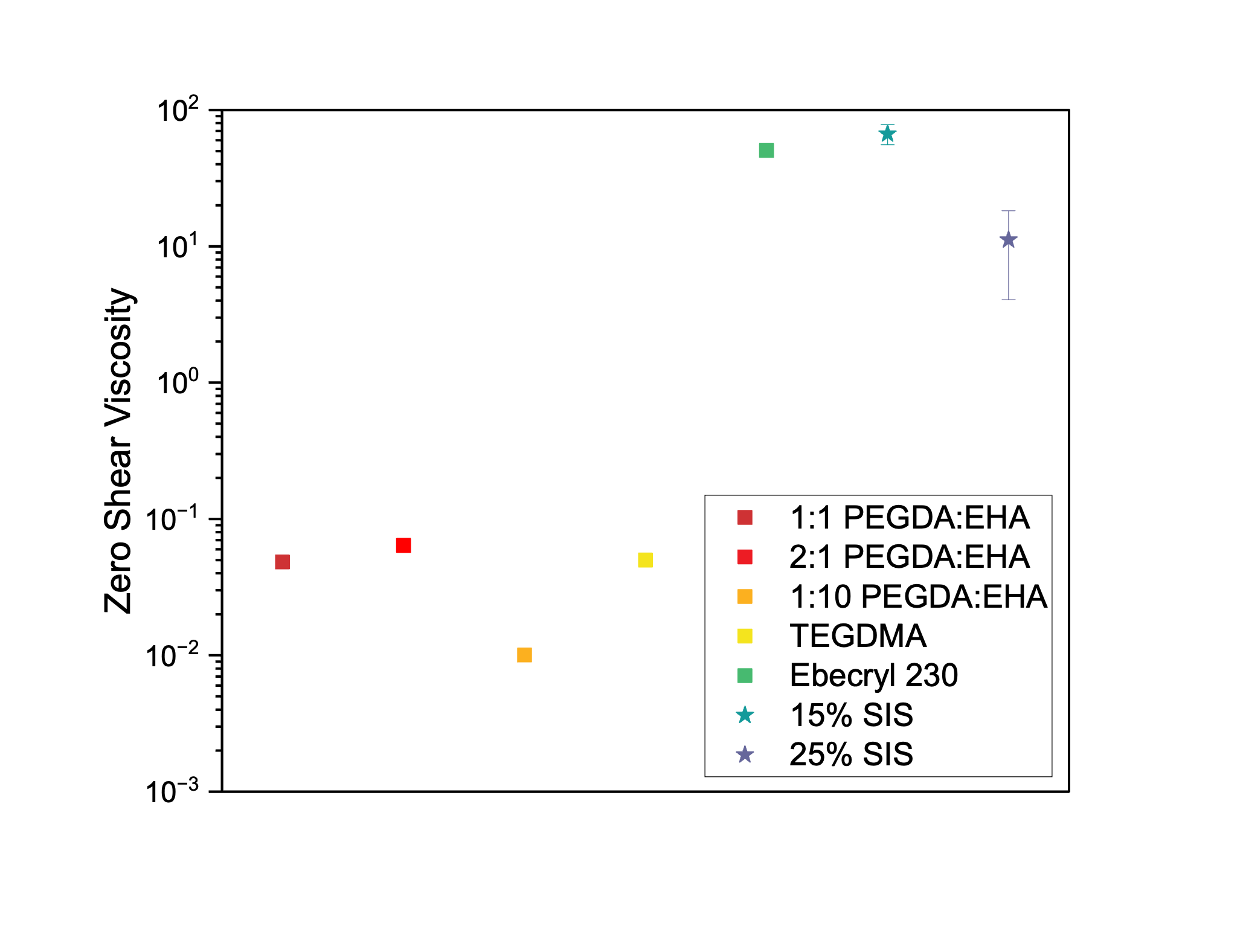


**Figure S3: Zero-shear viscosities of polymer binders used in ink formulations. Shear rate sweeps from** **0.01s^-1^ to 500s^-1^ were collected with an Anton Paar MCR 302 rheometer installed with a 25mm 2^°^ cone geometry. Zero-shear viscosity was extrapolated from linear plateau region at low shear rates.**

## S4. Shear Rate Sweeps for Ink Formulations


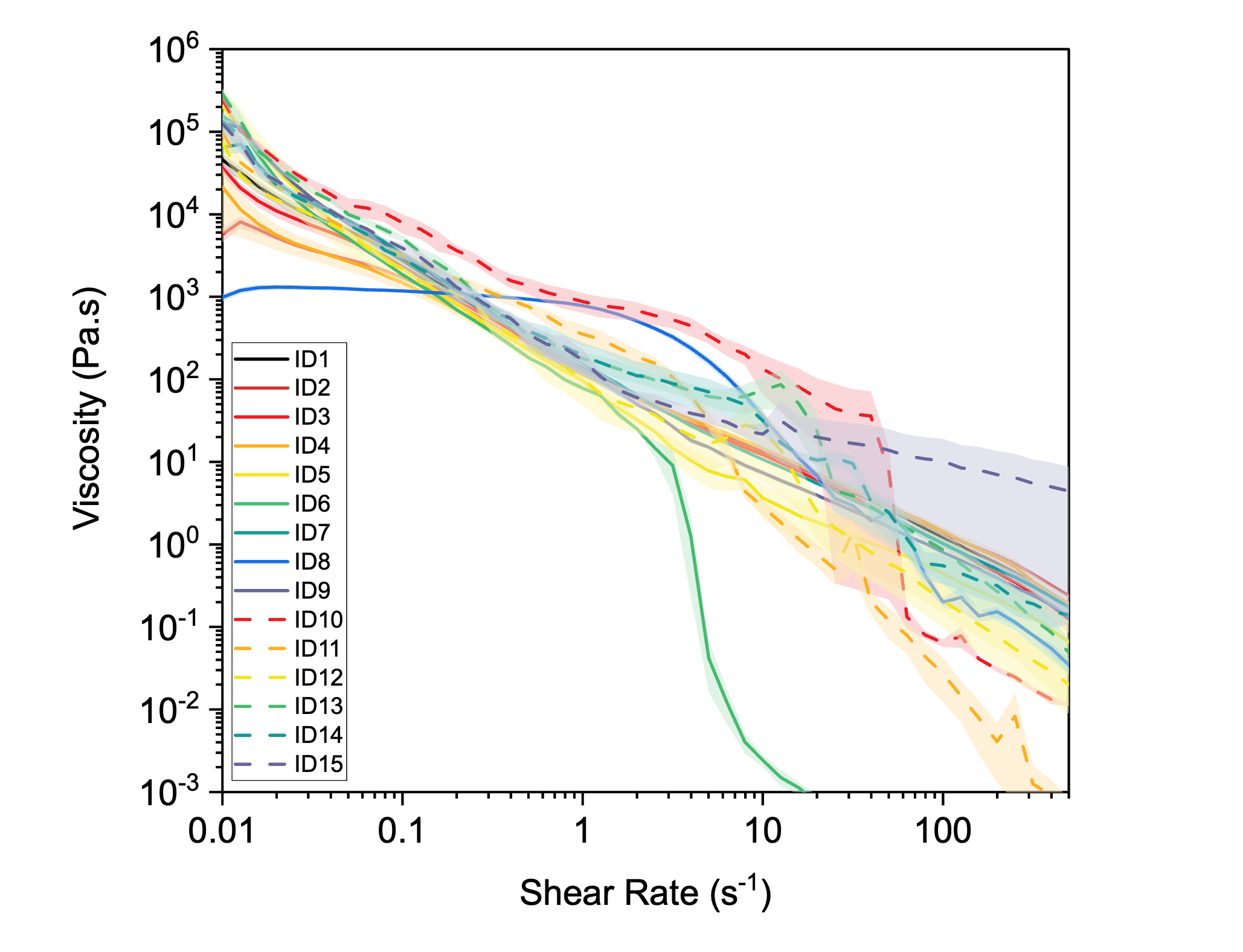


**Figure S4: Shear rate sweeps of each of the 15 formulations from 0.01s^-1^ to 500s^-1^. Samples ID6, ID10, and ID11 showed significant drops in viscosity due to loss of material at high shear rates.**
